# Quarantine Convention

**Published:** 1898-02

**Authors:** 


### EDITORIAL DEPARTMENT.

F. E. DANIEL, M. D., Editor.
S. E. HUDSON, M. D., Managing Editor.
A. J. SMITH. M. D., Galveston, Associate Editor.

Published Monthly at Austin, Texas, by Drs. Daniel and Hudson. Subscription price $2.00 a year in advance.

Eastern Representatives: Messrs. Monihan & Fairchild, St. Paul Building, 220 Broadway, New York City.

Official organ of the West Texas Medical Association, the Houston District Medical Association, the Austin District Medical Society, the Brazos Valley Medical Association, the Galveston County Medical Society, and several others.

### QUARANTINE CONVENTION.

The South, nay—the whole country—is worked up over the necessity of reform in quarantine measures.

The fact that despite good and efficient quarantine laws effectively administered in the Southern States shot-gun quarantine on the part of local authorities is not only possible but is resorted to,—makes some change necessary. The great question is, what shall that change be? In what shape shall the reform come?

As far as Texas is concerned a slight change in the existing law—which would put the question of local quarantine more completely in the hands of the State Health Officer—is all that is needed. As to other States we cannot say; but the writer is in favor of the preservation of State control of State quarantine, first, last and all the time.

There is tremendous pressure being brought to bear upon Congress in the interest of the Marine Hospital Service, and the great danger now threatening the country is that in their haste and want of consideration of facts, if not of a knowledge of facts Congress may put still greater powers—now, almost absolute, into the hands of this dangerous organization,—under the pretext that it is enlarging the powers of the Treasury Department. A blind man ought to see that the Secretary of the Treasury under the present law means the Surgeon-General of the Marine Hospital Service.

THE MARINE HOSPITAL SERVICE was organized for the care of sick mariners. Quarantine function was not thought of attaching to it, originally. But now it is likely to gobble up the whole quarantine system of the country, State and national.

Congress does not know, or will not believe, that the country is indebted to the inefficiency of the M. H. S. for three epidemics of yellow fever. It can be demonstrated that this service is directly responsible for the epidemic at Brunswick, Ga., in 1893, Biloxi in 1892 and Ocean Spiings in 1897.

It is now proposed, and two bills are before Congress to that end, to give the M. H. S., in the name of the Secretary of the Treasury, entire control of the question of quarantine, state, inter-state, national and inter-national; abrogating State rights entirely.

If the Southern States are not active and vigilant and take timely steps to defeat this scheme, it will be consummated before a year rolls by. The bill of Caffery was bad enough; that of Vest is worse.

Meantime our people, those who favor a Department of Public Health, and for the creation of which a bill has been prepared by the American Medical Association and endorsed by the American Public Health Association, the largest body of sanitarians in the world, are seemingly sleeping on their oars. Why has this bill not yet been introduced in Congress?

Meantime a petition has been sent to Congress, extensively signed by Southern health authorities, praying a delay in legislating on this subject. It will not be heeded. We will see; the Vest bill or the Caffery bill will be rushed through and become a law while our men are "considering."

For the purpose of getting a concensus of opinion as to what is best to be done to better protect the South from epidemic to lessen the evils that attach in some in instances to the present methods of quarantine

A BIG CONVENTION

Is called to meet at Mobile on the 9th of February, inst. At that convention not only the medical profession, but commerce, trade and travel will be represented.

The writer—the editor of the TEXAS MEDICAL JOURNAL, suggests, as a solution of the problem—how best to protect the South against yellow fever—abolish the M. H. S.—or make it *let State quarantine alone!* And we will be safe.

THE BILL creating a Public Health Department, as prepared by the American Medical Association, and which is to be presented to Congress, provides for a Department, and a Secretary of Public Health, to be appointed by the President. That is right—eminently fitting and proper.

But it also provides for an advisory council of some forty-five members, the heads of the respective State Public Health Departments. That feature is very objectionable. Such a body would be unwieldy. Before a meeting of such body could be gotten together the mischief would be done. Instead of such a council let there be but five members, chosen by the President or in some other way appointed. Let each State retain its autonomy; let the relation between State and nation be as it is in other matters.

The proposition to make the Secretary of the Treasury the head of the Public Health Department, or to tack on the great health interests to any other department, is simply idiotic. Push for a Department of Public Health.

